# Civilian moral injury: associations with trauma type and high-frequency heart rate variability in two trauma-exposed community-based samples

**DOI:** 10.1017/S003329172200215X

**Published:** 2023-08

**Authors:** Emma C. Lathan, Abigail Powers, Anna Kottakis, Alfonsina Guelfo, Greg J. Siegle, Jessica A. Turner, Matthew D. Turner, Vijwala Yakkanti, Jahnvi Jain, Yara Mekawi, Andrew P. Teer, Joseph M. Currier, Negar Fani

**Affiliations:** 1 Department of Psychiatry and Behavioral Sciences, Emory University School of Medicine, Atlanta, GA, USA; 2 Department of Psychiatry, University of Pittsburgh School of Medicine, Pittsburgh, PA, USA; 3 Department of Psychology, Georgia State University, Atlanta, GA, USA; 4 Department of Psychiatry and Behavioral Sciences, University of Texas Health Science Center at Houston, Houston, TX, USA; 5 Department of Psychological and Brain Sciences, University of Louisville, Louisville, KY, USA; 6 Department of Psychology, University of South Alabama, Mobile, AL, USA

**Keywords:** Civilians, heart rate variability, moral injury, sexual violence, trauma exposure

## Abstract

**Background:**

Moral injury exposure (MIE) and distress (MID) may indirectly affect the relationship between trauma exposure and alterations in autonomic regulation [assessed via high-frequency heart rate variability (hfHRV)] in civilians, but this has not been tested in prior research. We conducted two exploratory studies to examine trauma types' associations with MIE and MID among civilian medical patients (Study 1) and explore how these facets may indirectly affect the relationship between trauma type and hfHRV among civilians seeking mental health services (Study 2).

**Methods:**

Participants recruited from a public hospital and/or community advertisements (Study 1, *n* = 72, 87.5% Black, 83.3% women; Study 2, *n* = 46, 71.7% Black, 97.8% women) completed measures assessing trauma type, MIE, and MID. In Study 1, trauma types that emerged as significant correlates of MIE and MID were entered into separate linear regression analyses. Trauma types identified were included as predictors in indirect effects models with MIE or MID as the mediator and resting hfHRV (assayed via electrocardiography) as the outcome.

**Results:**

Childhood sexual abuse emerged as the only significant predictor of MIE, *b* = 0.38, *p* < 0.001; childhood sexual abuse, *b* = 0.26, *p* < 0.05, and adulthood sexual assault, *b* = 0.23, *p* < 0.05 were significant predictors of MID. Participants with greater MIE and MID demonstrated lower hfHRV. Adulthood sexual assault showed an indirect effect on hfHRV through MID, *B* = −0.10, s.e. = 0.06, 95%CI (−0.232 to −0.005).

**Conclusions:**

Moral injury was uniquely associated with sexual violence and lower hfHRV in civilians. Data highlight moral injury as a pathway through which autonomic dysregulation may emerge and its salience for trauma treatment selection.

Exposure to traumatic events, or situations that involve actual or threatened death, serious injury, or sexual violence, is an established risk factor for various physical and mental health conditions including chronic pain, cardiovascular disease, and posttraumatic stress, depressive and anxiety disorders, among others (Kessler et al., 2017; Liu, Petukhova, & Sampson, 2017). The onset, severity, and course of trauma-related outcomes, however, are largely dependent on the ways in which a traumatic event is appraised (Woud, Verwoerd, & Krans, 2017). Indeed, trauma appraisals, defined as the cognitive interpretations of or meaning assigned to trauma, often better predict psychological symptoms than the event itself (DePrince, Chu, & Pineda, 2011; Lathan, Selwyn, & Langhinrichsen-Rohling, 2021; Martin, Cromer, DePrince, & Freyd, 2013), suggesting how an individual thinks about or processes the event can foster or impede recovery. As such, negative trauma appraisals, such as self-blame, guilt, shame, or betrayal, are thought to play a crucial role in the development and maintenance of post-trauma pathology (Woud et al., 2017; Woud, Kleim, & Cwik, 2019).

Moral injury, or a sense of one's moral or ethical code being violated, is a relatively new and evolving construct that has been conceptualized as a type of trauma appraisal (Hoffman & Nickerson, 2021). Moral injury is characterized by severe psychological distress and functional impairments that may occur after experiencing, witnessing, perpetrating, or failing to prevent stressful events that transgress core values and moral beliefs (McEwen, Alisic, & Jobson, 2021); namely, distinct psychological symptoms, such as guilt, shame, self-blame, disgust, betrayal, and existential issues, may arise when an individual experiences a traumatic event that conflicts with their deeply held moral beliefs about themselves, others, and the world (Litz et al., 2009).

Although moral injury often co-occurs with posttraumatic stress disorder (PTSD), recent clinical and neurobiological data support moral injury as a unique, transdiagnostic construct (Barnes, Hurley, & Taber, 2019; Sun et al., 2019) that essentially involves transgressions of an individual's moral beliefs and expectations, i.e., their conceptualization of ‘what's right and wrong.’ Moral injury is often preceded by an event that evokes later dissonance regarding the situation and the morality of the actions taken by the self or others. For example, if a person views themselves as benevolent and altruistic, the moral injury may develop if the individual directly contributed (even unintentionally) to an event that endangered the life or well-being of another person. The moral injury was originally conceptualized to better understand service members and veterans' combat-related experiences (e.g. perpetrating violence, being betrayed by a trusted authority figure) and resultant symptoms (e.g. guilt, shame, betrayal, disgust) that were not fully captured by existing PTSD criteria (Drescher et al., 2011; Litz et al., 2009). As such, moral injury has been primarily studied in military populations to date (Currier, Drescher, & Nieuwsma, 2021).

More recently, researchers have begun to assess the moral injury and its effects in civilian populations. Fani et al. (2021) found that greater trauma exposure across the lifespan was linked to greater exposure to morally injurious events (i.e. moral injury exposure, MIE) and the presence of moral injury distress (MID) in a chronically trauma-exposed community sample; a history of childhood maltreatment was associated with MIE, while both childhood maltreatment and interpersonal trauma in adulthood were associated with MID (Fani et al., 2021). Findings provide preliminary support for MIE and MID as distinct components of moral injury development among civilians (Currier et al., 2017; Currier, Holland, Rojas-Flores, Herrera, & Foy, 2015; Fani et al., 2021). While a growing body of literature suggests greater cumulative trauma exposure is related to higher levels of moral injury among trauma-exposed civilians (Currier et al., 2015; Fani et al., 2021; Hoffman, Liddell, Bryant, & Nickerson, 2018), no prior study has examined the associations between specific trauma types, MIE, and MID. A more thorough understanding of which trauma types best predict MIE and MID is needed to better detect and target moral injury and its effects during patient encounters with trauma-exposed civilians.

Deleterious outcomes, including suicide attempts, anger, and symptoms of PTSD, anxiety, depression, and burnout, have been noted across civilians who endorse MIE and MID (Currier et al., 2015; Hoffman et al., 2018; Nickerson et al., 2015, 2018; Plouffe et al., 2021). Going beyond psychological symptoms, a crucial trauma-related outcome that remains unstudied in relation to moral injury is autonomic nervous system (ANS) dysfunction. The ANS comprises parasympathetic and sympathetic nervous systems, which work in conjunction to maintain homeostasis by regulating the cardiovascular, respiratory, and digestive systems and allow adaptive adjustment to internal and external stimuli (Jänig, 2022). It is well-established that trauma exposure disrupts the rhythmic balance between the parasympathetic and sympathetic branches of the ANS (Schauer & Elbert, 2010) such that activation of the parasympathetic branch, which is responsible for restful and adaptive emotion regulatory responses, is suppressed (Gillie & Thayer, 2014).

High-frequency heart rate variability (hfHRV) is a physiological marker of parasympathetic nervous system tone (Berntson et al., 1997; Berntson, Quigley, & Lozano, 2007) that reflects the fluctuation in the length of intervals between heart beats (Kim, Cheon, Bai, Lee, & Koo, 2018). Low resting hfHRV has been associated with greater vulnerability to physical and psychological stressors and disease, including cardiovascular disorders and inflammation (Alen, Parenteau, Sloan, & Hostinar, 2021; Kim et al., 2018; Kubota, Chen, Whitsel, & Folsom, 2017). Low resting hfHRV has been consistently associated with greater trauma exposure (Liddell et al., 2016) and PTSD (Gillie & Thayer, 2014; Schneider & Schwerdtfeger, 2020); alterations in hfHRV in adults have the strongest associations with early life maltreatment or associated psychological factors (e.g. emotion dysregulation), as opposed to psychological diagnoses *per se* (Meyer et al., 2016).

The ability to contextualize experiences by using adaptive appraisals is related to reduced levels of psychophysiological distress (Schartau, Dalgleish, & Dunn, 2009). Previous findings link shame and blame appraisals with low HRV and other markers of autonomic arousal (Freed & D'Andrea, 2015; León, Hernández, Rodríguez, & Vila, 2008), suggesting moral injury appraisals may be a key target for psychological intervention. Given established connections between hfHRV and both trauma and other appraisals, it is possible that specific trauma types (e.g. interpersonal trauma) are indirectly associated with lower hfHRV through the effects of MIE and MID. However, to our knowledge, physiological correlates of MIE and MID have not yet been explored, including racially marginalized individuals (e.g. Black individuals), who may experience moral injury more frequently due to witnessing and directly experiencing community violence and racial injustice. Further, Black individuals have also been historically excluded from psychophysiological research (Bradford et al., 2022). Given the heterogeneity in neurobiological responses to trauma (Schmidt, Kaltwasser, & Wotjak, 2013) and how psychophysiological profiles (i.e. hfHRV) associated with PTSD appear to predict response to first-line trauma treatments (Thomaes et al., 2014), it is important to assess whether moral injury may have an indirect impact on these responses; moral injury is a potential mechanism through which autonomic dysregulation occurs among trauma-exposed civilians, but this possibility has not yet been tested. If the moral injury is a mechanism for the development of autonomic dysregulation in the aftermath of trauma, it merits attention as a trauma treatment target.

## Study 1 and study 2

Given the paucity of research on moral injury occurring among civilian trauma survivors, particularly among individuals of color, this exploratory study examined trauma-related and physiological correlates (specifically, hfHRV) of MIE and MID in two trauma-exposed, community-based samples that predominantly include Black Americans, extending extant research in two distinct ways. Study 1 extends the findings of Fani et al. (2021) by examining which type(s) of traumatic events are most predictive of the exposure and distress components of moral injury in a sample of majority Black American civilians seeking medical care from an urban, publicly funded (i.e. safety net) hospital. We hypothesized that interpersonal traumas would be most strongly associated with MIE and MID, given our prior research (Fani et al., 2021).

Study 2 explores associations between moral injury and hfHRV in a demographically similar sample of civilians (majority Black American) seeking mental health services for PTSD. We hypothesized that higher MIE and MID would be associated with lower hfHRV. Where significant associations were observed between trauma type and MIE and MID in Study 1, we examined these trauma types as predictors of hfHRV in Study 2 and tested for indirect effects of MIE and/or MID on the association between trauma types and hfHRV.

## Study 1 method

### Participants and procedure

Participants (*n* = 72; *M*_age_ = 40.6 years; s.d._age_ = 12.5 years) were primarily Black (90.3%, *n* = 65), women (84.7%, *n* = 61), high school graduates (or equivalent; 84.7%, *n* = 61), and experiencing significant economic disadvantage (65.2%, *n* = 47; i.e. reported a monthly household income of less than $2000). See Table 1 for sample characteristics. Participants were recruited from a large, publicly funded healthcare system in Atlanta, Georgia for involvement in an ongoing study of trauma exposure and related symptoms in underserved, primarily Black communities Grady Trauma Project. As part of the larger study, trained experimenters approached patients in medical clinics regarding potential participation. During the COVID-19 pandemic, patients with medical appointments were contacted via telephone and invited to participate. The experimenters verbally administered a variety of self-report measures to consenting individuals. All procedures contributing to this work comply with the ethical standards of the relevant national and institutional committees on human experimentation and with the Helsinki Declaration of 1975, as revised in 2008. Informed consent was obtained from all study participants after the nature of the procedures was explained. The MIE and MID data of 39 Study 1 participants (54%) have been previously published elsewhere (Fani et al., 2021).

**Table 1.** Study 1 and Study 2 sample characteristics

Variable	Study 1 (*n* = 72) %	Study 2 (*n* = 46) %
Gender		
Woman	84.7	97.8
Man	13.9	0.0
Missing	1.4	2.2
Race/Ethnicity		
Black/African American	90.3	71.7
White	4.2	19.6
Multiracial	1.4	4.3
Other	4.2	2.2
Missing	–	2.2
Education Level		
Less than 12th grade	15.3	10.9
High school graduate or equivalent	38.8	13.0
Some college or technical school	23.6	19.6
College or technical school graduate	13.9	30.4
Graduate school	8.3	23.9
Missing	–	2.2
Current employment status		
Unemployed	68.1	47.8
Employed	31.9	50.0
Missing	–	2.2
Household monthly income		
$0–249	8.3	13.0
$250–499	8.3	4.3
$500–999	16.7	19.6
$1000–1999	31.9	15.2
⩾ $2000	26.4	43.5
Missing	8.3	4.3
Mean age	40.6 years (s.d. = 12.5)	43.2 years (s.d. = 11.4)

### Measures

#### Trauma exposure

The Traumatic Events Inventory (TEI; Gillespie et al., 2009; Mekawi et al., 2021) was used to assess participants' self-reported exposure to 14 different types of traumatic events. Each item was rated on a binary scale (0 = *No*, 1 = *Yes*). Six trauma type variables were generated by collapsing similar traumas into broad binary categories: experiencing childhood abuse (three items; e.g. physical, sexual, emotional), sexual assault in adulthood (one item), witnessing violence (six items), experiencing physical assault by a non-partner (two items), experiencing intimate partner violence (IPV; two items), and exposure to a serious accident, injury, or illness (three items). For example, if a participant endorsed witnessing an attack with or without a weapon, witnessing violence between caregivers, witnessing the murder of a family member or friend, they were coded as having witnessed violence (0 = *Did not witness violence*, 1 = *Witnessed violence*). A cumulative trauma exposure variable was generated by summing the total number of trauma types endorsed.

#### Moral injury

The Moral Injury Exposure and Symptom Scale – Civilian (MIESS-C; Fani et al., 2021) is a 10-item self-report questionnaire measuring moral injury development across two subscales: MIE (five items; e.g. ‘I saw things that were morally wrong’) and MID (five items; e.g. ‘I am troubled because I violated my morals by failing to do something that I felt I should have done’). In the current study, participants were briefed with the following, ‘In this scale we will be asking you about events that might have conflicted with your morality or your sense of right and wrong.’ Responses ranged from 1 (*Strongly Disagree*) to 6 (*Strongly Agree*). Internal consistencies of the MIESS-C subscales were in the acceptable range (MIE, *α* = 0.71; MID, *α* = 0.65; Ursachi, Horodnic, and Zait, 2015).

#### Data analyses

Seventy-five individuals were assessed for inclusion in the current study. Of these, three individuals denied exposure to one or more of the trauma types assessed, and therefore, were removed from additional analyses. Thus, 72 participants comprised the Study 1 sample, though sample sizes differ by analyses based on the availability of data. Per trauma type, between 1.4% and 2.8% of all cases had missing data (*n* = 1–2). Only one participant (1.4%, *n* = 1) had missing data on the MID variable. Because missing data were minimal, they were handled via mean imputation.

Pearson's, point-biserial, and phi-coefficient correlations were conducted to determine bivariate associations among trauma types, MIE, and MID, examined as different families of tests. Bonferroni correction was applied to correct for error due to multiple comparisons; statistical significance was set at *p* < 0.008 for each family of tests. Trauma types that emerged as significant correlates of MIE and MID were entered into two separate linear regression analyses with MIE and MID serving as the dependent variables. Given the exploratory nature of this study, statistical significance was set at *p* < 0.05 for regression analyses.

## Study 1 results

All participants (*n* = 72) reported exposure to at least one of six trauma types [e.g. experiencing childhood abuse (i.e. physical, sexual, emotional), experiencing sexual assault in adulthood, witnessing violence, experiencing physical assault by non-partner, experiencing IPV, exposure to a serious accident, injury, or illness; *M*_types_ = 3.7; s.d._types_ = 1.5]. Notably, the majority of participants endorsed witnessing violence (81.9%, *n* = 59), exposure to a serious accident or injury (73.6%, *n* = 53), experiencing childhood abuse (66.7%, *n* = 48), experiencing physical assault by someone other than a romantic partner (61.1%, *n* = 44), and experiencing IPV (55.6%, *n* = 40). See Table 2 for descriptive statistics.

**Table 2.** Study 1 and Study 2 descriptive statistics

Variable	Study 1 (*n* = 72)			Study 2 (*n* = 46)		
	% Endorsed			% Endorsed		
Trauma type						
Experienced childhood abuse	66.7%			84.4%		
Physical	30.6%			43.2%		
Sexual	50.0%			60.0%		
Emotional	44.4%			61.4%		
Experienced adulthood sexual assault	22.2%			49.0%		
Witnessed violence	81.9%			86.7%		
Experienced non-partner violence	61.1%			42.2%		
Experienced IPV	55.6%			48.9%		
Exposure to accident/injury/illness	73.6%			82.2%		
	Min – Max	*M*	s.d.	Min – Max	*M*	s.d.
Moral injury						
Exposure	10–30	20.78	5.57	5–29	18.30	5.78
Distress	7–30	18.78	6.22	5–30	17.02	6.63
Resting hfHRV	–	–	–	−1.40–0.80	−0.06	0.45

Presence/absence of childhood abuse, *r_pb_*(72) = 0.45 *p* < 0.001, was positively associated with MIE; this relationship remained significant following correction for multiple comparisons. See Table 3 for bivariate associations. As such, we conducted a follow-up regression analysis to examine the strength of associations of MIE with specific types of childhood abuse (i.e. physical, sexual, emotional) as predictors. Regression analysis revealed a significant overall model, *F*_(3,68)_ = 9.63, *p* < 0.001, accounting for 29.8% of the variance in MIE. Childhood sexual abuse, *b* = 0.38, *p* *<* 0.001, emerged as the only significant predictor of MIE.

**Table 3.** Study 1 Pearson's, point biserial, and phi-coefficient correlations

Variable	2	3	4	5	6	7	8
1. Moral injury exposure	0.87***	0.45***	0.27	0.24	0.27	0.04	0.03
2. Moral injury distress	--	0.34**	0.36**	0.21	0.27	0.04	0.00
3. Experienced childhood abuse	--	--	0.30	0.29	0.18	0.35**	0.16
4. Experienced adulthood sexual assault	--	--	--	0.05	0.21	0.33**	0.09
5. Witnessed violence	--	--	--	--	0.31**	0.18	0.08
6. Experienced non-partner assault	--	--	--	--	--	0.23	0.09
7. Experienced IPV	--	--	--	--	--	--	0.02
8. Exposure to accident/injury/illness	--	--	--	--	--	--	--

**p ⩽ 0.008, ***p ⩽ 0.001.

Presence/absence of adulthood sexual assault, *r_pb_*(72) = 0.36, *p* = 0.002, and childhood abuse, *r_pb_*(72) = 0.34, *p* = 0.004, were both positively correlated with MID; these relationships remained significant following correction for multiple comparisons. A follow-up regression analysis was conducted to examine the strength of associations between MID, adulthood sexual assault, and childhood abuse types (i.e. physical, sexual, emotional). Regression analysis revealed a significant overall model, *F*_(4,67)_ = 5.02, *p* < 0.001, accounting for 23.1% of the variance in MID. Childhood sexual abuse, *b* = 0.26, *p* < 0.05, and adulthood sexual assault, *b* = 0.23, *p* < 0.05, emerged as significant predictors of MID.

## Study 2 method

### Participants and procedure

Participants (*n* = 46; *M*_age_ = 43.2 years; s.d._age_ = 11.4 years) were primarily Black (71.7%, *n* = 33), women (97.8%, *n* = 45), high school graduates (or equivalent; 89.1%, *n* = 41), and experiencing significant economic disadvantage (52.2%, *n* = 24; i.e. reported a monthly household income of less than $2000). Participants were enrolled in a PTSD intervention study (Fani et al., 2019) involving electrocardiogram data collected at rest; several of these participants were recruited via the urban hospital and community advertisements. The Study 1 and Study 2 samples were independent; none of the Study 1 participants was included in Study 2. Specific data collection and study procedures can be found in Study 1. MIE and MID data from 30 Study 2 participants (65%) have been previously published elsewhere (Fani et al., 2021).

All participants reported exposure to two or more of six types of trauma [e.g. experiencing childhood abuse (i.e. physical, sexual, emotional), experiencing sexual assault in adulthood, witnessing violence, experiencing physical assault by non-partner, experiencing IPV, exposure to a serious accident, injury, or illness; *M*_types_ = 3.9; s.d._types_ = 1.3]. The trauma types most frequently endorsed included witnessing violence (84.8%, *n* = 39), experiencing childhood abuse (82.6%, *n* = 38), and exposure to a serious accident, injury, or illness (80.4%, *n* = 37). See Table 2 for descriptive statistics.

### Measures

See Study 1 Method section for information on the measurement of trauma exposure and moral injury.

#### Electrocardiography (ECG)

ECG data were collected during intervention sessions to derive measures of hfHRV from a two-lead montage (right neck, left wrist; 2000 Hz, no filters), using a BIOPAC ECG100C Electrocardiogram amplifier (BIOPAC Systems, Inc) via their AcqKnowledge software version 4.0 (BIOPAC System, Inc).

*Task design.* During the first intervention visit, participants sat in a chair in a sound-attenuated chamber in front of a computer screen and microphone, while being monitored by a researcher. Instructions appeared on the screen, which instructed them to either engage in breath focus (10 trials, half of which were augmented with breath-synchronized vibration) or rest (five trials), each for one min. These conditions were randomized throughout a 15-min session to yield five one-min ‘rest’ segments (Baek, Cho, Cho, & Woo, 2015), which were analyzed in the current manuscript.

*ECG Processing.* ECG data were exported to text files which were processed and cleaned using via custom Matlab code. R-wave peaks were extracted and converted to inter-beat interval series. Series were subjected to continuous Morelet waveform transforms to yield a task-wise running (100 Hz) estimate of power in the hfHRV band (0.18–0.4 Hz) using the HRV-AS package (Ramshur, 2010, available at https://github.com/jramshur/HRVAS). Further information on processing techniques used can be found in Rabellino et al. (2017).

Quality assurance metrics included: heart rate between 40 and 120 beats per minute; no significant abrupt spikes or ectopic beats; electrocardiograms that appear normative with distinct R peaks and QRS complexes. Data that were flagged on quality assurance metrics (e.g. HR outside the 40–120 beats per minute range) were manually checked and corrected by hand-marking missed beats and hand-deleting erroneous heart-beat detections. Hand-deletion was relevant for eight participants, whose baseline electrocardiogram data were found to be unusable.

Mean hfHRV was calculated within each of the rest trials and averaged across these trials for the first assessment day per participant. hfHRV data were Winsorized (i.e. data outside the Tukey Hinges of 1.5 times the interquartile range from the 25th and 75th percentiles were rescaled to the last good value within the Tukey Hinges) and entered into group analyses.

### Data analyses

For trauma type, 2.2 to 4.3% of all cases had missing data (*n* = 1–2). Eight participants' baseline electrocardiogram data (17.4%) were unusable due to noise. Mean imputation was used to handle missing-ness for trauma type and hfHRV.

Pearson's bivariate correlations were conducted to examine associations among MIE, MID, and hfHRV. Hayes' SPSS PROCESS Macro was used to conduct follow-up analyses (Zhao, Lynch, & Chen, 2010). Trauma type(s) that emerged as significant predictors of MIE and MID via Study 1 were included in Study 2 analyses to better understand relations with hfHRV. Separate mediation models were conducted. In each model, trauma type was entered as the predictor, MIE or MID as the mediator, and hfHRV as the outcome. In each model (*n* = 46), unstandardized indirect effects were computed for each of 5000 bootstrapped samples. Bonferroni correction was applied to correct for error due to multiple comparisons; statistical significance was set at *p* < 0.025 for each family of tests.

## Study 2 results

### Moral injury exposure

**Fig. 1.**
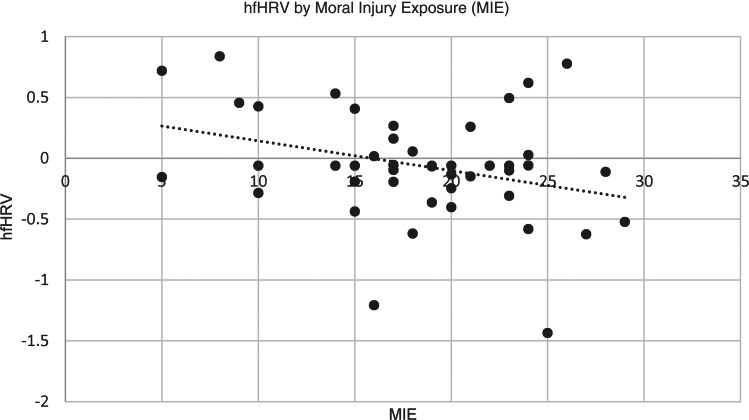
Scatterplot of hfHRV by moral injury exposure (MIE).

As expected, results of bivariate correlations revealed that MIE was associated with lower hfHRV at the bivariate level, *r*(46) = −0.31, *p* < 0.05 (See Fig. 1). The indirect effects model revealed that childhood sexual abuse was not significantly associated with MIE, *B* = 0.48, s.e. = 1.77, *p* = 0.78. MIE was significantly associated with decreased hfHRV, *B* = −0.03, s.e. = 0.01, *p* = 0.03. No significant indirect effect of childhood sexual abuse on hfHRV was found, *B* = −0.01, s.e. = 0.05, 95% CI (−0.104 to 0.086).

### Moral injury distress

**Fig. 2.**
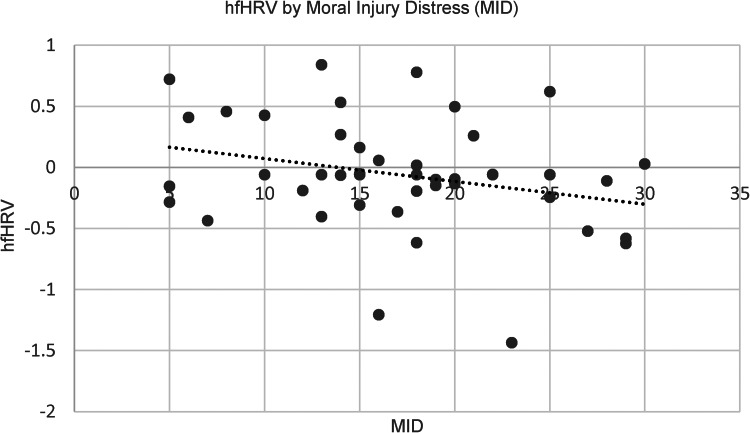
Scatterplot of hfHRV by moral injury distress (MID).

**Fig. 3.**
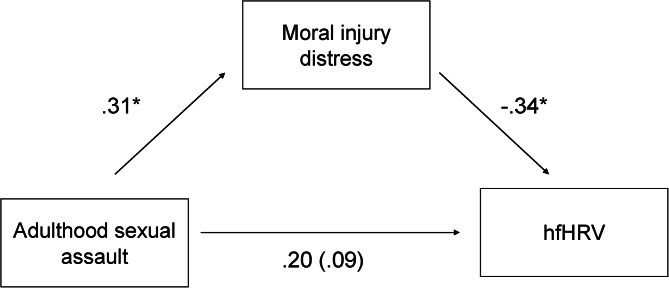
Simple mediation model.

MID was not significantly correlated with lower hfHRV at the bivariate level, *r*(46) = −0.28, *p* = 0.06 (See Fig. 2). When included in an indirect effects model, adulthood sexual assault was positively associated with MID, *B* = 4.17, s.e. = 1.90, *p* = 0.03, and MID was associated with decreased hfHRV, *B* = −0.02, s.e. = 0.01, *p* = 0.03. There was a small indirect effect from adulthood sexual assault to hfHRV via MID after correction for multiple comparisons, *B* = −0.10, s.e. = 0.06, 95% CI (−0.232 to −0.005) (See Fig. 3). In the second model, childhood abuse did not significantly predict MID, *B* = 1.31, s.e. = 2.03, *p* = 0.52. However, MID significantly predicted hfHRV, *B* = −0.02, s.e. = 0.01, *p* = 0.05. There was no significant indirect effect of childhood sexual abuse on hfHRV *B* = −0.03, s.e. = 0.04, 95% CI (−0.140 to 0.044).

## General discussion

The current studies examined relations among the exposure and distress components of moral injury, trauma type, and autonomic response in trauma-exposed civilians. In line with our first hypothesis, Study 1 found that exposure to certain traumatic events was tied to greater endorsement of moral injury appraisals, with slightly differing patterns across the MIE and MID components of moral injury development. Study 2 demonstrated that participants with higher MIE and MID demonstrated proportionally lower hfHRV, indicating that moral injury is linked to significant autonomic dysregulation in civilians. Follow-up analyses revealed that there was a nominal indirect effect from adulthood sexual assault to low hfHRV via MID, suggesting identifying and addressing distress appraisals may help disrupt the established pathway from sexual assault to low hfHRV among trauma-exposed Black women (Lee & Theus, 2012).

Regarding specific trauma types, experiencing childhood abuse was correlated with MIE, while sexual assault in adulthood was correlated with both MIE and MID. Witnessing violence, experiencing physical assault by a non-partner, experiencing intimate partner violence, and exposure to serious accidents, injuries, or illnesses were related to neither MIE nor MID in this population. Follow-up analyses revealed that, when compared to all other trauma types assessed, childhood sexual abuse had the strongest association with MIE, and childhood sexual abuse and sexual assault in adulthood had stronger associations with MID.

Our results suggest that sexual violence may be a particularly potent risk factor for moral injury appraisals, especially distress related to morally injurious events. This may be, in part, due to the established relations between sexual violence and the core features of moral injury, such as guilt, shame, existential conflict, and mistrust (Jinkerson, 2016). More broadly, it is possible that the relationship between sexual violence and moral injury is influenced by the internalization of rape culture (i.e. widespread acceptance of rape myth beliefs, or misconceptions about sexual assault that shift the blame from perpetrator to victim), as survivors tend to blame themselves for the assault, feel ashamed, and question their own morality and dignity (Bhuptani & Messman-Moore, 2019). Because trauma appraisals can influence the onset, severity, and course of mental health outcomes, future research should examine the roles survivors' broad beliefs about sexual assault and particular negative cognitions about the traumatic event play in MIE and MID after sexual violence.

Moreover, sexual violence across the lifespan was differentially related to moral injury appraisals. In Study 1, experiencing childhood sexual abuse was related to both MIE and MID, while sexual assault in adulthood was related to MID. Therefore, it is possible that the risk for exposure and distress varies with the developmental stage. It is important to note that very little is known about the relations between sexual violence and moral injury, so the mechanisms underlying these lifespan differences remain unclear. It has been hypothesized, however, that moral injury has a ‘kindling effect with respect to trauma’ (Fani et al., 2019, p. 7); perhaps, sexual violence-related moral distress increases over time. In Study 2, however, childhood sexual abuse was related to neither MIE nor MID, although the relationship between adulthood sexual assault and MID observed in Study 1 remained. Altogether, adulthood sexual assault seems to be a more reliable correlate of moral injury across populations than childhood sexual abuse. Current results, in conjunction with similar findings (Fani et al., 2021), highlight the importance of further exploring if, how, and why sexual violence across the lifespan differentially relates to MIE and MID.

In addition, as most of our sample were Black women, it is likely that these participants have experienced other forms of pervasive violence and injustice – namely, racial trauma (Department of Justice, 2014). This term has been used to describe ongoing individual racial discrimination and systematic marginalization (Comas-Díaz, Hall, & Neville, 2019) that is experienced more frequently by Black Americans than any other racial group (Anderson & Stevenson, 2019). Because racial trauma is a particularly salient trauma type for Black Americans, it may be particularly relevant to their perceptions of MIE and MID. Experiences of gendered racial discrimination may be especially harmful for individuals with intersectional identities, such as Black women (Moody & Lewis, 2019). Further, recent evidence suggests an inverse effect between discrimination and resting HRV among African American students (Hill et al., 2017). While present findings advance our knowledge of how childhood maltreatment and adult sexual trauma are associated with MIE and MID among Black Americans, there is much left to be learned about moral injury's potential role in the relationship between racial trauma and hfHRV.

Moreover, racially marginalized women experience disproportionate rates of sexual assault and PTSD (Asnaani & Hall-Clark, 2017; Gluck et al., 2021; Merrick, Ford, Ports, & Guinn, 2018); further, many of these individuals report experiencing moral injury, and MIE has been associated with emotion dysregulation (Ray, Hunsanger, Nagy, & Pickett, 2021) and past suicide attempt (Fani et al., 2021). However, little attention has been paid to the role of moral injury appraisals in functional impairment in the aftermath of trauma, and similarly, they are often ignored as a treatment target. While further investigation is warranted, results indicate a systematic assessment of moral injury in trauma-exposed civilians may help inform trauma-focused treatment selection and targets. For example, a mental health provider may consider Trauma Informed Guilt Reduction (TrIGR) therapy (Norman et al., 2019) or Adaptive Disclosure (Gray, Binion, Amaya, & Litz, 2021) in addition to more traditional PTSD treatments (i.e. Cognitive Processing Therapy, Prolonged Exposure) when a client endorses experiencing one or more of the core components of moral injury.

Current results should be interpreted in light of specific study limitations, including reliance on self-report measures for trauma history as well as cross-sectional methodology, which bars causal conclusions. Underlying causal relationships may exist, such that the relationships are reversed or reciprocal, or an unmeasured factor confounds findings. Moreover, our studies' limited sample sizes (Study 1, *n* = 75; Study 2, *n* = 46) may have prevented statistically significant findings from being detected (i.e. type II error) given insufficient statistical power. The internal consistencies of MIE and MID were in the acceptable range but lower than expected. Additional research on the psychometric properties of the MIESS-C is needed to inform best practices related to the assessment of moral injury appraisals.

Further, the study measured hfHRV using five one-min intervals, which occurred randomly within a 15-min breath-focus task. While guidelines for hfHRV research generally recommend five-minutes of contiguous data, one-min intervals are considered acceptable (Baek et al., 2015), particularly within the context of the employed wavelet analytic technique, which assesses hfHRV continuously throughout the task (Laborde, Mosley, & Thayer, 2017); the extent to which our resting assessments also incorporate recovery from previous task-blocks or anticipation of future task-blocks is unclear. Moving forward, further research examining moral injury's associations with other markers of autonomic function, such as pupil diameter and galvanic skin response, is warranted to obtain a more comprehensive understanding of the impact of moral injury on autonomic regulation.

Future research may benefit from attempting to replicate the current studies in larger treatment-seeking and community-based samples with a longer contiguous resting-state assessment. Additionally, our findings reflect the study populations, two groups of primarily low-income, trauma-exposed, Black women seeking physical or mental health treatment in the southeastern United States. Although results add to the limited number of studies examining hfHRV among Black women, it is possible that our results may not generalize to other race and gender civilians (e.g. white individuals, men) or in other geographic regions, non-treatment seeking civilians, and those who are insured or seeking care at private hospitals.

Despite noted limitations, these findings contribute to the literature by providing the first examination of moral injury's relations with trauma type and hfHRV among civilians. We found strong links between moral injury, adulthood sexual assault and autonomic disruptions among two samples of primarily Black women seeking health care. Overall, our data support the notion that targeting moral injury appraisals via culturally responsive, trauma-informed psychological interventions may help to reduce autonomic dysfunction, particularly among Black women with histories of sexual violence.
